# Supplementary material for: Deletion of wfs1 Impairs Oligodendrocyte Precursor Cells Dorsal Distribution and Myelination Through the wfs1-hmgcs1 Axis in Zebrafish
Source: Biology (Basel). 2026 Aug 16;15(16):1402. doi: 10.3390/biology15161402 (PMC13510151; doi:10.3390/biology15161402)
Supplement: Supplementary file 1 [file biology-15-01402-s001.zip › File S1-Transcriptome sequencing data and differentially expressed genes (DEGs) in wfs1-- zebrafish at 5 dpf/2.KEGG/wfs1KOvsWT/DOWN/src/dre04270.html]

dre04270


- dre:337226

- Down regulated genes

337226(-2.98693262894109)

- dre:497384

- Down regulated genes

497384\_1(-2.05267237809407)

- dre:436718

- Down regulated genes

436718\_1(-6.85571323128554)

- dre:492486

- Down regulated genes

492486\_1(-1.65362606223537)

- dre:337226

- Down regulated genes

337226(-2.98693262894109)

- dre:406370

- Down regulated genes

406370\_1(-1.88308518916857)
- dre:100148818

- Down regulated genes

100148818(-1.62621488580639)

- dre:566430

- Down regulated genes

566430\_1(-3.52930553579608)

- dre:436966

- Down regulated genes

436966(-1.74302137366672)

- dre:403114

- Down regulated genes

403114(-2.18938533161417)

Close
